# The Impact of Health Investment on Industrial Structure: “Spillover” or “Crowding Out”?—Evidence From Emerging Market Countries

**DOI:** 10.3389/fpubh.2021.833961

**Published:** 2022-01-24

**Authors:** Qiao Chen, Hai-Ming Wei, Yu-Peng Zhi

**Affiliations:** ^1^School of Tourism and Hotel Management, Hubei University of Economics, Wuhan, China; ^2^College of Economics and Management, Nanning Normal University, Nanning, China; ^3^School of Economics, Guangxi University, Nanning, China

**Keywords:** health investment, upgrading of industrial structure, emerging market countries, non-linear relationship, spillover or crowding out

## Abstract

Using national dynamic panel data from 21 emerging markets between 1999 and 2020 and bidirectional fixed effect and threshold regression methods, this paper evaluated the impact of health investment on industrial structure upgrading from two aspects of economic output and economic structure. The results showed that: (1) public health investment and private health investment have a crowding out effect on the added value of primary and secondary industries, and the crowding out effect of public health investment is greater than that of private health investment; (2) Public health investment and private health investment have a spillover effect on the added value of the tertiary industry, and the spillover effect of public health investment is greater than that of private health investment; (3) Both public and private health investment contribute to the transfer of the labor force to the tertiary industry, and tests showed the baseline regression results were robust and reliable; (4) The relationship between health investment and industrial structure upgrading was non-linear. As per capital GDP increases, the inhibition effect of public health investment on industrial structure upgrading gradually weakens whilst the promotion effect of private health investment on industrial structure upgrading gradually increases. The results of this study clarify how health investment affects industrial structure, and offers new guidance for health investment policy formulation in emerging market countries.

## Introduction and Literature Review

According to the World Health Organization, strokes and heart attacks caused by working long hours caused ~813,000 deaths worldwide in 2019, an increase of nearly 31 percent since 2000. The majority occurred in emerging market countries where rapid industrialization has created environmental pollution, economic poverty and psychological stress. Consequently emerging market countries are characterized by below average per capital GDP and above average rates of disease mortality (1). Such countries are subject to the dilemma of “disease before wealth.” In recent years, with the rapid development of artificial intelligence technology and increasingly aging populations, the tension between achieving health and economic growth goals in emerging markets has rapidly intensified, especially between investment in health or industrial structure upgrades (2). In the theory of human capital, health is an important basic component. If investment in health is reduced, the health of the population deteriorates, which affects the quality of human capital and reduces labor productivity. This in turn impacts the potential for industrial structure upgrades. Increases in health investment improve the health of the population and reduce the depreciation rate of human capital, which is conducive to demographic dividends. However, the level of investment in other industries is reduced, thus inhibiting economic growth. This study aims to clarify the impact of health investment on industrial structure and determine whether different types of health investment have different effects on industrial structure upgrades. The nature of the relationship between public (government) and private (household) health investment on industrial structure at different per capital GDP levels is also explored.

Early scholars focused on the relationship between human capital investment (including population health, education and infrastructure investment) and sustainable economic growth. Fisher pointed out “Report on the Health of the Nation” presented to the U.S. Congress, health is a national asset, and increased investment in health reduces rate of disease and contributes to economic growth. However, he did not explain the specific mechanisms by which health investment promotes economic growth (3). In 1961, Schultz put forward the concept of human capital, formed by investments in education, health and immigration. This is an innovative analysis of human capital in health (4). Mushkin formally proposed health as a component of human capital, developing the concept of healthy human capital. Education and health were regarded as the two products of human capital (5). From then on, scholars have carried out a large number of theoretical and empirical studies on the relationship between health investment and economic growth.

The literature related to this research topic can be divided into two categories: One is about the negative correlation between health investment and economic growth or industrial structure upgrading. Weisbrod pointed out that health investment is human capital investment, healthy food consumption promotes the improvement of human nutrition level, and is one of the main reasons for promoting economic growth (6). Narayan et al. regarded health as a type of lasting capital that could be continuously produced, whereby investment in health would increase with age, crowding out investment in material capital and inhibiting economic development, neither of which are not conducive to industrial structure upgrades (7). Mohapatra and Mishra found a causal relationship between health investment and GDP over the short and long term, in which excessive health investment stifled growth (8). Lv found that large amounts of public health expenditure inhibited economic development in developed countries, although the level of inhabitation varied depending on the level of national economic development and national health quality (9). The other is the research on the positive correlation between health investment and economic growth or industrial structure upgrading. Research on the relationship between health and economic structure initially focused on analyzing the industrial nature of health investment. Chakravarthi et al. and Guan et al. both found that the expansion of health investment and health services was conducive to the development of the tertiary industry (10, 11). Yao et al. found that the impact of public health investment on the proportion of the employed population in the service industry was less than that of private health investment (12). However, she did not find an clear crowding out effect of public or private health investment on the manufacturing industry. Wangwe et al. believe that public health will promote economic structural transformation (13). Arawomo et al. point out that health investment will not immediately promote economic growth in the short term, and has a crowding out effect on physical capital investment (14). López et al. analyzed the impact of human capital accumulation and human capital structure on per capita income and found a positive correlation between human capital accumulation and per capita income (15). Lu and Zou found that health human capital has a significant positive effect on residents' income, and the government can narrow the income gap by adjusting public health investment policies (16). Other researchers have suggested that if public health spending is sufficient, it can offset the negative impact of public health spending on economic growth in developing countries (17, 18). Zhang and Xia found that health investment influence economic growth through five channels: population, savings, education, labor, leisure and technological progress (19). In addition, some studies showed health investment limited economic growth in the short term due to the reduction of material capital. But over the long term, health investment increased the human capital of the labor force, which supported economic growth.

In general, the effect of health investment on economic growth has been widely researched, although there are still some deficiencies. Firstly, most of the existing literature focuses on the relationship between health investment and economic growth and there are relatively few studies on its relationship with industrial structure upgrading. Second, most studies have examined the linear relationship between health investment and economic structure but have ignored the non-linear relationship between health investment and industrial structure upgrading under different GDP levels. Therefore, from the perspective of the theory of healthy economic growth, this paper analyses the relationship between health investment and industrial structure upgrading. Furthermore, the mechanisms by which public and private health investment have inhibitory and spillover effects on healthy economic growth are clarified. Using data from emerging market countries, relevant conclusions are drawn from empirical tests, and policy suggestions are put forward.

The paper makes several contributions to the literature. Firstly, the effect of health investment on industrial transformation and upgrading in emerging market countries is discussed. Secondly, It enriches the research content of health investment. The relative impacts of public (government) health investment and private (family) health investment are delineated. Thirdly, the non-linear relationship between health investment and industrial structure upgrading is discussed. Fourthly, the mechanism of health investment influencing industrial structure upgrading through human capital and material capital is discussed. The results provide useful insights for emerging market countries. The rest of the paper is arranged as follows. Sections Model Setting and Variable Descriptions and Data Sources and Descriptive Statistics describe the data, variables and empirical model used. Section Empirical Analysis outlines the results of the data analysis. The results are discussed in section Discussion before offering conclusions in section Summary and Conclusions.

## Model Setting and Variable Descriptions

### Measurement of Industrial Structure Upgrading

Following previous studies (20–22), the upgrading of industrial structure was selected as a proxy variable for the upgrading of industrial institutions. The ratio of the added value of the tertiary industry to the added value of the secondary industry is used as the index of industrial structure upgrading, as shown in the following equation:


(1) $$\begin{array}{l} {Indu_{-}Sen}_{it}\;=\;\frac{{{{Indu}_{it}}_{-}T}_{it}}{{{Indu_{it}}_{-}S}_{it}} \end{array}$$

Where the value of _*Ind*_*u*_*it*_−_*Fit*_,_*Ind*_*u*_*it*_−_*Sit*_ and _*Ind*_*u*_*it*_−_*Tit*_ represent the added value of the primary, secondary and tertiary industries respectively; Primary industry mainly refers to the production of food materials and some other biological materials. Second industry mainly refers to the processing and manufacturing industry. Tertiary industry refers to industries other than the primary and secondary industries, such as transportation, communications, commerce, etc. *Indu*_*s*_−_*Senit*_ indicates the upgrading of industrial structure. It is expressed by the ratio of the added value of the tertiary industry to the added value of the secondary industry.

### Two-Way Fixed Effects Model

The impact of health investment on industrial structure upgrading not only has individual effect but also time effect. Therefore, this paper used a bidirectional fixed effects model as the benchmark regression model. Details are as follows:


(2) $$\begin{array}{l} {Indu_{-}Sen}_{it}\;=\;\alpha_{0}\;+\;\alpha_{1}{Heal_{-}Nat}_{it}\\ +\;\alpha_{2}{Heal_{-}Pri}_{it}\;+\;\alpha_{3}Control_{it}\;+\;\mu_{it} \end{array}$$

where *i* represents one of the 21 emerging market countries, *t* represents the year, and μ_*it*_ represents random a disturbance term. *Indu*_*it*_ reflects the proportion of added value of each industry in GDP. Health investment is the core explanatory variable which is represented by public health (*Healt*_*h*_−_*Natit*_) investment and private health (*Healt*_*h*_−_*Priit*_) investment. Following Yao et al. (12), government medical and health expenditure and per capital medical and health expenditure of residents were used as measurement indicators (23).

### Threshold Regression Model

Although the linear regression model can measure the impact of healthy investment on industrial structure upgrading. However, under different per capita income levels in emerging market countries, is there any difference between health investment and industrial structure upgrading? The question is still worth exploring. Threshold regression model can reveal the effect of multiple independent variables on a dependent variable, and measure the non-linear relationship between health investment and industrial structure upgrading in different per capita income levels. So considering the possible non-linear relationship between health investment and industrial structure upgrading, a panel threshold regression model was constructed. Starting with the basic single threshold panel model, the model was set as follows:


(3) $$\begin{array}{l} y_{it}\;=\;u_{i}\;+\;\beta_{1}x_{it}I(q_{it}\leq \gamma )\;+\;\beta_{2}x_{it}I(q_{it}\;>\;\gamma )\\ \;+\theta X_{it}\;+\;\varepsilon_{it} \end{array}$$

where *q*_*it*_ is the threshold variable representing per capital GDP, *I*(·) is an indicative function: when *q*_*it*_ ≤ γ, *I*(·) = 1; When *q*_*it*_ > γ, *I*(·) = 0; γ is the unknown threshold value, and $\varepsilon_{it}\sim iid\left(0,\delta^{2}\right)$ is the random disturbance term.


(4) $$y_{it}\;=\;\{\begin{array}{c} u_{i}\;+\;\beta_{1}x_{it}\;+\;\theta X_{\text{it}}\;+\;\varepsilon_{it}\;,\;q_{it}\leq \gamma \;\\ u_{i}\;+\;\beta_{2}x_{it}\;+\;\theta X_{\text{it}}\;+\;\varepsilon_{it}\;,\;q_{it}>\gamma \; \end{array}$$

The model is a piecewise function, where the coefficient of *x*_*it*_ is β_1_ when *q*_*it*_ > γ, and β_2_ when *q*_*it*_ > γ. A multiple threshold model was then constructed as follows:


(5) $$\begin{array}{r} y_{it}\;=\;u_{i}\;+\;\beta_{1}x_{it}I\left(q_{it}\;\leq \;\gamma_{1}\right)\;+\;\beta_{2}x_{it}\\ I\left({\gamma_{1}\;<\;q}_{it}\;\leq \;\gamma_{2}\right)\;+\;\beta_{3}x_{it}I\left(q_{it}\;>\;\gamma_{2}\right)\;+\;\theta X_{\text{it}}\;+\;e_{it} \end{array}$$

The “grille search method” proposed by Hansen 1999 was used to create the candidate threshold γ_*i*_ in the threshold regression. 0.0025 was taken as the grille level, and the range of candidate threshold values were graded (24). The candidate threshold value γ_*i*_ corresponding to the square and minimum value of the model residual, *S*_1_(γ), was selected as the required true threshold value $\hat{\gamma }$ bearing.

A threshold effect test was used to test whether there was a significant difference between β_1_ and β_2_ in the regression results. The constraint condition β_1_ = β_2_ was applied to the regression model corresponding to the established threshold value, and then the Wald test was performed (25). If the confidence probability of the Wald statistic was < 0.05, the null hypothesis was rejected. If there is a significant difference between β_1_ and β_2_, the threshold effect is significant (26).

An authenticity test was used to investigate whether the threshold estimate obtained by the test was equal to its true value. The null hypothesis, *H*_0_ *posits that γ* = γ_0_. Hansen (24) proposed a maximum likelihood estimator to test the threshold value. The corresponding likelihood ratio statistic is defined as follows:


(6) $$\begin{array}{l} LR\left(\gamma \right)\;=\;\left(SSR_{1}\left(\gamma \right)\;-\;SSR_{1}\left(\hat{\gamma }\right)\right)/\delta^{2}\left(\hat{\gamma }\right) \end{array}$$

where *SS*_1_(γ) is the sum of squared residuals obtained after parameter estimation under the null hypothesis. It was assumed that the variance of the residual error could be obtained after parameter estimation under the original assumption $\delta^{2}\left(\hat{\gamma }\right)$. Referring to Hansen's (24) study, when $LR\left(\gamma \right)\;>\;-2log{\left(1-(1\;-\;a)\right)}^{\frac{1}{2}})$ is rejected, the critical value of the *LR* statistic is 7.35.

*Control*_*it*_ represents a set of control variables. Per capital GDP (*PG*_*it*_) was used as a proxy for economic development level. Total capital formation (*GC*_*it*_) was used to measure the investment rate, reflecting the impact of dynamic capital accumulation on industrial structure. The level of foreign investment (*FI*_*it*_) was used as a proxy for the degree of open border trading, so that its influence on industrial structure could be analyzed. The labor resources (*PR*_*it*_), defined as a proxy for labor supply. Finally, the unobserved effects of region *i* and time *t* were controlled.

## Data Sources and Descriptive Statistics

Unbalanced panel data from 21 emerging market countries were selected for empirical analysis. The sample period was from 1999 to 2020, and the data such as _*d*_*u*_*it*_−_*Fit*_,_*d*_*u*_*it*_−_*Sit*_ and _*d*_*u*_*it*_−_*Tit*_ were obtained from Penn World Table 9.1 and the World Bank database. *CS*_*it*_ stands for GDP per capita, reflecting the level of economic development, *GC*_*it*_ stands for total capital formation, *FI*_*it*_ represents net foreign direct investment, which reflects a country's level of openness,*PR*_*it*_ represents the number of people of working age, which reflects the level of Labor supply in a country. The average growth rates of government public investment and family private investment in emerging market countries are 11.59 and 58.45% respectively, while the average real GDP growth rate is about 4% (The World Bank). Some countries even have negative growth. Table 1 shows the descriptive statistics of the variables used in this study, such as industrial added value, number of employees, and the industrial structure advanced index of emerging market countries. Each variable had obvious heterogeneity, which met the requirements of the econometric model.

**Table 1:** Descriptive statistics of variables.

**Variable**	**Obs**	**Mean**	**Std. Dev**.	**Min**	**Max**
*Ind*_*u*_−_ *Senit*_	884	6.94	6.388	0.471	36.212
*Ind*_*u*_*it*_−_ *F*	959	32.25	6.57	16.953	61.844
*Ind*_*u*_*it*_−_ *S*	959	14.383	10.276	1.541	54.919
*Ind*_*u*_*it*_−_ *T*	884	48.295	8.403	22.308	88.724
*Emp*_*l*_*it*_−_ *F*	570	24.497	6.347	13.345	45.419
*Emp*_*l*_*it*_−_ *S*	570	24.891	15.562	2.636	62.562
*Emp*_*l*_*it*_−_ *T*	570	50.612	12.4	18.9	72.292
*Hea*_*l*_−_ *Natit*_	361	458.199	418.917	18.312	2514.624
*Hea*_*l*_−_ *Priit*_	361	310.779	193.886	55.216	1133.509
*CS*_*it*_	1009	3385.819	4150.45	53.537	23494.596
*GC*_*it*_	1016	23.952	7.35	4.527	95.32
*FI*_*it*_	809	−6708	204300	−2317000	416700
*PR*_*it*_	1140	65.494	17.144	36.49	105.758

## Empirical Analysis

### Benchmark Regression of the Impact of Health Investment on Industrial Added Value

**Figure 1:**
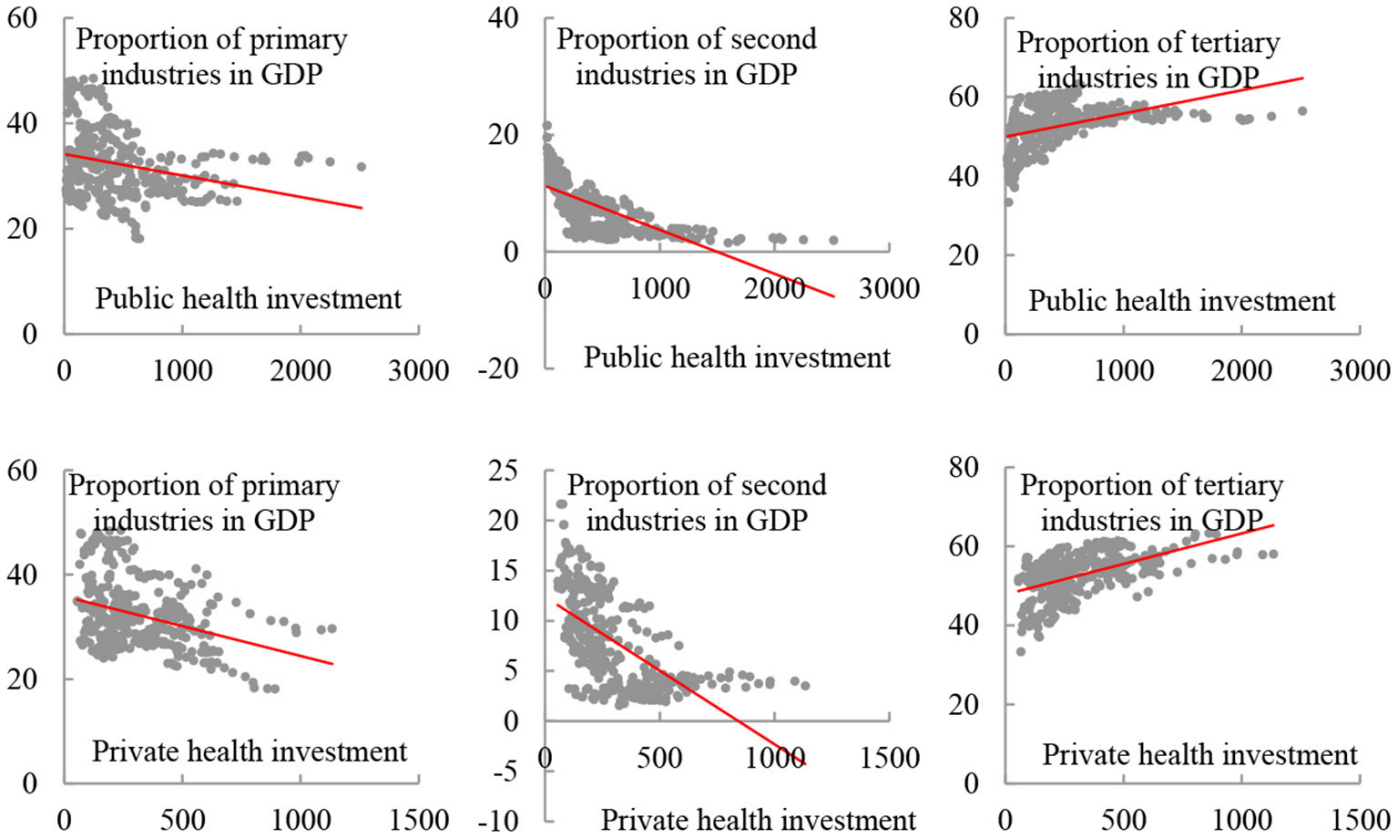
Scatter charts illustrating the levels of health investment and added value to GDP by each industry.

Figure 1 shows the impacts of the two types of health investment on the growth of primary, secondary and tertiary industries respectively. Both public and private health investment were significantly negatively correlated with the proportion of added value of primary and secondary industries to GDP. It can be inferred that public health investment and private health investment crowd out the primary and secondary industries. The proportion of added value of the tertiary industry to GDP was positively correlated with health investment (27, 28). There was a spillover effect on the tertiary industry. Although the scatter charts (Figure 1) illustrate the correlation between health investment and added value of various industries, this conclusion needs to be further verified by scientific measurement methods.

There was a significant negative correlation between both public and private health investment and the added value of the primary industry (Table 2). When public health investment increased by 1%, industrial added value decreased by 0.09%. Similarly when private health investment increased by 1%, industrial added value decreased by 0.08%. Simultaneous increases in public and private health investment had a larger inhibitory effect on primary industry of −1.30 and −1.19 respectively. With an increasingly aging population, it is probable that the demographic dividend will disappear completely, labor resources will decrease, and the crowding out effect of health investment on the primary industry will gradually appear.

**Table 2:** Baseline regression results.

**Variable**	_****Indu**_**it**_ **−****_ **F**			_****Indu**_**it**_ **−****_ **S**			_****Indu**_**it**_ **−****_ **T**		
	**(1)**	**(2)**	**(3)**	**(4)**	**(5)**	**(6)**	**(7)**	**(8)**	**(9)**
*Hea*_*l*_−_ *Natit*_	−0.09^***^		−1.30^***^	−0.01		−0.24^**^	0.09^***^		1.52^***^
	(−3.94)		(−5.34)	(−1.01)		(−2.16)	(3.70)		(6.03)
*Hea*_*l*_−_ *Priit*_		−0.08^***^	−1.19^***^		−0.01	−0.22^**^		0.08^***^	1.40^***^
		(−3.46)	(−4.99)		(−0.82)	(−2.08)		(3.17)	(5.70)
*PG*_*it*_	−0.46^**^	−0.48^***^	−0.43^**^	−0.46^***^	−0.46^***^	−0.45^***^	0.74^***^	0.76^***^	0.70^***^
	(−2.57)	(−2.67)	(−2.46)	(−5.87)	(−5.91)	(−5.81)	(3.94)	(4.04)	(3.89)
*GC*_*it*_	0.13^***^	0.13^***^	0.16^***^	−0.07^***^	−0.08^***^	−0.07^***^	−0.10^**^	−0.10^**^	−0.14^***^
	(3.11)	(2.96)	(3.87)	(−4.03)	(−4.09)	(−3.73)	(−2.29)	(−2.14)	(−3.15)
*FI*_*it*_	−0.33^**^	−0.33^**^	−0.32^**^	0.08	0.08	0.08	0.20	0.19	0.18
	(−2.59)	(−2.54)	(−2.58)	(1.48)	(1.49)	(1.53)	(1.47)	(1.43)	(1.42)
*PR*_*it*_	0.09^**^	0.10^***^	0.05	0.06^***^	0.06^***^	0.05^***^	−0.15^***^	−0.16^***^	−0.09^**^
	(2.58)	(2.77)	(1.30)	(4.00)	(4.08)	(3.35)	(−3.91)	(−4.10)	(−2.49)
C	29.20^***^	20.58^***^	149.96^***^	7.33^***^	6.40^***^	29.85^***^	57.63^***^	66.01^***^	−85.17^***^
	(10.55)	(7.88)	(6.15)	(6.10)	(5.67)	(2.74)	(19.92)	(24.17)	(−3.38)
*N*	361	361	361	361	361	361	361	361	361
*R* ^2^	0.71	0.71	0.72	0.72	0.72	0.72	0.76	0.76	0.76

***, ** and * *represent significance levels of 1, 5 and 10% respectively*.

There was also a significant negative correlation between both public and private health investment and the added value of the secondary industry. When public health investment increased by 1%, the added value of secondary industry decreased by 0.01%. When private health investment increases by 1%, the added value of secondary industry decreased by 0.01%. When public and private health investment increased simultaneously, the inhibitory effect on secondary industry increased significantly to −0.24 and −0.22, respectively.

The results showed a significant positive correlation between both public and private health investment and the tertiary industry. The impact coefficients of public and private health investment on the tertiary industry were 0.09 and 0.08 respectively, and when both were increased, the influence effect significantly increased to 1.52 and 1.40 respectively. The remaining control variables passed the significance test. It can be seen that public and private health investment both inhibit the growth of added value in the primary and secondary industries, creating a significant crowding out effect, while public and private health investment promotes the growth of added value in the tertiary industry, creating a significant spillover effect.

It may be that the government's health investments have a hindering effect on the growth of primary and secondary industries by crowding out the accumulation of material capital. In the short term the total amount of capital investment is unlikely to change. When the government taxes health investments to a certain extent, excessive investment in health will place a greater financial burden on the government, and it will dilute the government's investment in other industries, which will increase the government's financial burden and force the government to reduce investment in primary and secondary industries. Then it forms different degree of extrusion effect. Health investment itself belongs to the tertiary industry, so both public health investment and private health investment have significant spillover effects on the tertiary industry. Health investment covers not only labor-intensive industries (e.g. elderly care and medical services) (29), but also technology-intensive industries (e.g. medical drugs and medical devices) (30). Therefore, higher levels of medical investment increase the national demand for health services and related products, thus promoting the value-added growth of the tertiary industry.

Health investment has spillover effects on the tertiary industry by increasing the stock of healthy human capital. Increased health investment in emerging market countries can extend the life expectancy and health of the population, which improves productivity and ensures the scale of labor supply in the tertiary industry (31, 32). Increasing investment in health also stimulates the demand for health services and related products, which attracts more workers to health services and related industries, thereby promoting the growth of the tertiary industry. Zhu et al. (2019) pointed out that health investment can expand the scope of health services. It is beneficial to the development of the tertiary industry to increase the number of health workers and reduce the pressure on hospitals by turning technical medical services into social preventive services (33).

### Robustness Test

We have analyzed the relationship between health investment and primary, secondary, and tertiary industries in emerging market countries, using a two-way fixed effects model. However, these conclusions may be subject to measurement errors and endogeneity. To verify the reliability of the model setting and regression results, the explained variables were adjusted to test the robustness of the model. In the existing literature industrial added value is usually measured using a substitute index of the ratio of employment to total employment. In this paper the proportion of people employed in each of the primary, secondary and tertiary industries was used as a measure of the added value of each industry. Table 3 shows the regression results of the employment ratio proxy variable.

**Table 3:** Robustness test.

**Variable**	_****Empl**_**it**_ **−****_ **F**			_****Empl**_**it**_ **−****_ **S**			_****Empl**_**it**_ **−****_ **T**		
	**(1)**	**(2)**	**(3)**	**(4)**	**(5)**	**(6)**	**(7)**	**(8)**	**(9)**
*Hea*_*l*_−_ *Natit*_	−0.04^**^		−0.03^**^	−0.23^***^		−2.04^***^	0.17^***^		0.19^***^
	(−2.26)		(−1.97)	(−8.30)		(−7.17)	(8.09)		(9.63)
*Hea*_*l*_−_ *Priit*_		−0.01^***^	−0.01^**^		−0.21^***^	−1.78^***^		0.01^***^	0.01^***^
		(−2.63)	(−2.38)		(−7.61)	(−6.39)		(6.67)	(8.39)
*PG*_*it*_	0.60	0.34	0.99^**^	−1.94^***^	−1.97^***^	−1.89^***^	2.24^***^	1.25^***^	0.79^***^
	(1.58)	(1.40)	(2.42)	(−9.06)	(−9.10)	(−9.32)	(13.74)	(4.96)	(3.48)
*GC*_*it*_	0.17^***^	0.18^***^	0.16^***^	−0.18^***^	−0.19^***^	−0.13^***^	0.01	0.12^***^	0.06^*^
	(5.62)	(5.97)	(5.28)	(−3.46)	(−3.65)	(−2.76)	(0.17)	(3.11)	(1.65)
*FI*_*it*_	−0.44^***^	−0.47^***^	−0.47^***^	0.09	0.10	0.11	0.32^***^	0.23^*^	0.31^***^
	(−4.85)	(−5.17)	(−5.17)	(0.60)	(0.63)	(0.76)	(2.73)	(1.91)	(2.91)
*PR*_*it*_	−0.03	−0.05^**^	−0.04	0.26^***^	0.27^***^	0.19^***^	−0.24^***^	−0.24^***^	−0.18^***^
	(−1.10)	(−2.11)	(−1.56)	(5.93)	(6.20)	(4.41)	(−7.10)	(−7.07)	(−5.58)
C	22.10^***^	23.37^***^	23.39^***^	27.57^***^	5.40^*^	208.71^***^	53.94^***^	57.61^***^	46.02^***^
	(12.90)	(13.03)	(13.10)	(8.33)	(1.72)	(7.32)	(21.37)	(23.48)	(18.52)
*N*	361	361	361	361	361	361	361	361	361
*R* ^2^	0.26	0.27	0.27	0.62	0.61	0.66	0.66	0.65	0.72

*, ** and *** *indicate significance at 10, 5 and 1% levels respectively*.

Following substitution of the proxy variable, the influence coefficient of both public and private health investment were consistent with the baseline regression. The influence coefficient of public health investment and private health investment on employment in primary and secondary industries is negative, but the influence coefficient on employment in tertiary industry is positive. That is, public and private health investment crowded out the number of people employed in the primary and secondary industries, and increased the number of people employed in the tertiary industry. Public health investment also had a greater effect on employment than private health investment. Increasing public and private health investment attracts more workers to engage in health services and related labor-intensive industries, which may stimulate the transfer of labor resources from the primary and secondary industries to the tertiary industry, thus promoting employment growth in the tertiary industry (34, 35). In addition, compared with public health investment, private health investment in emerging market countries is more conducive to increasing employment in the tertiary industry, and private health investment has less crowding out effect on employment in the secondary industry. This means that strengthening private health will create more jobs in the tertiary sector and have a less negative effect on employment in the secondary industry. The possible reason is that, compared with public health investment, private medical services tend to be diversified, flexible, less external, and more beneficial to individual patients. Thus, more opportunities for employment and value creation can be provided. The form of public health investment is relatively simple, which leads to its employment and value creation capacity is lower than private health investment. The above results show that the model regression results do not change after the substitution of proxy variables, which suggests the results are robust and reliable.

## Discussion

The results showed that health investment had a significant spillover effect on the output value of the tertiary industry and the proportion of people it employed. It also had a significant crowding out effect on the output value of the primary and secondary industries (36, 37). Whether this influence effect changes a country's industrial structure or promotes industrial structure upgrades is discussed in the following sub-sections. Furthermore, we discuss whether there is a non-linear relationship between health investment and industrial structure upgrading at different per capital GDP levels.

### The Mechanism of Health Investment on Industrial Structure

**Figure 2:**
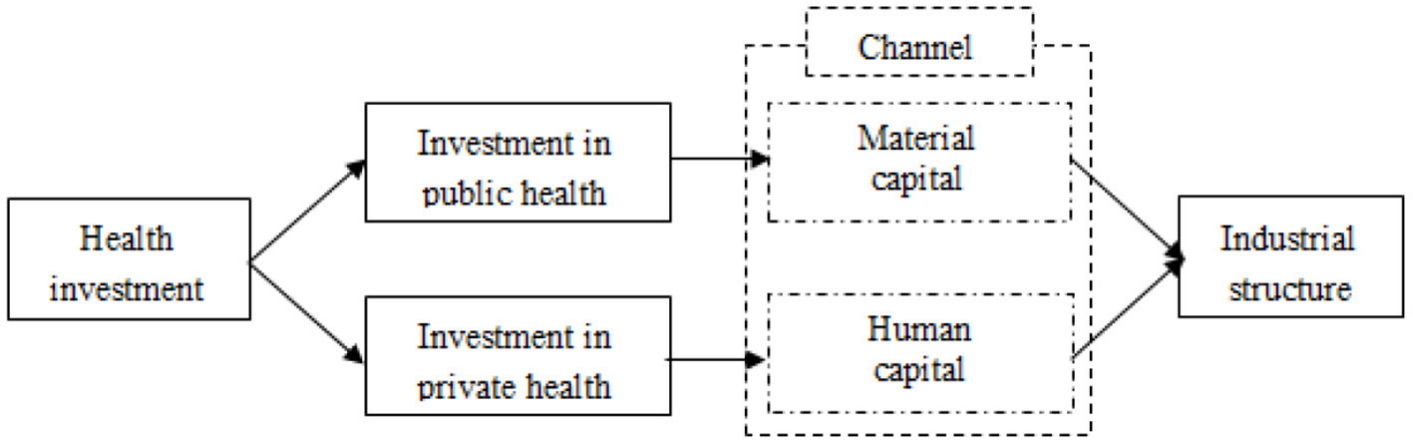
The influence mechanism of health investment on industrial structure.

Health investment affects industrial structure mainly through two channels (Figure 2). On the one hand, health investment affects industrial structure through material capital channel. As for public health investment, public health investment inhibits industrial structure by crowding out material capital accumulation. When the government taxes for health investment to a certain extent, excessive health investment makes the government bear high costs, resulting in capital dilution, crowding out material capital investment, slowing down economic growth, and not conducive to the upgrading of industrial structure (38). However, for private health investment, it has the advantages of diversified forms, high flexibility, low externality, higher benefit degree of individual patients and so on. It can improve market vitality and promote capital flow. Therefore, private health investment has spillover effect on industrial structure upgrading. On the other hand, health investment affects industrial structure through human capital channel. Health investment promotes economic growth by increasing the stock of healthy human capital (39). Both public and private investment in health can improve the health level and life expectancy of citizens, thus increasing the country's health human capital and promoting economic growth and industrial structure upgrading. At present, countries in the world are gradually aging, resulting in the shortage of young and middle-aged cheap labor force, the labor cost advantage of low-end manufacturing industry is gradually losing, and the era of unlimited supply of labor force is gone forever. Economic development gradually from “labor chasing capital” to “capital chasing labor” (40). Under the mediating effect of healthy human capital and material capital, the net effect of health investment on industrial structure upgrading depends on the size of the two mechanisms.

### The Impact of Public Health Investment on the Upgrading of Industrial Structure

The significance of four three capital GDP thresholds (single, double or triple thresholds) at which public health investment led to industrial structure upgrades were tested to determine the form of the model. The *F* statistic and *P* value of each model were estimated using a “self-sampling method” (Table 4). The results showed that the effect of the single and double thresholds was significant at the 5 and 1% level respectively, while the effect of a triple threshold was not significant. As a result, the following analysis will be based on the double threshold model.

**Table 4:** Effect test of public health investment thresholds.

**Model**	**Critical value**					
	***F* value**	***P* value**	**BS times**	**1%**	**5%**	**10%**
Single threshold	165.761^***^	0.005	500	157.652	106.780	75.549
Double thresholds	129.647^**^	0.020	500	146.235	97.877	70.781
Triple thresholds	−136.261	0.365	500	−55.011	−73.301	−81.519

*** and ** *represent significance levels of 1 and 5 % respectively*.

The two threshold values in the double threshold model were then estimated using a likelihood ratio function (Figure 2). Estimated public health investment (*Hea*_*l*_−_*Natit*_) thresholds are equivalent to the value of γ when *LR* is 0. As can be seen from Table 5, the threshold regression model of health investment and industrial structure upgrading has a double threshold effect, and the estimated thresholds ${\hat{\gamma }}_{1}$ and ${\hat{\gamma }}_{2}$ were 4,412 and 12,000 respectively.

**Table 5:** Public health investment threshold estimates and confidence intervals.

	**Threshold estimate**	**95% confidence interval**
Threshold value ${\hat{\gamma }}_{1}$	4,412	[3,694, 4,499]
Threshold value ${\hat{\gamma }}_{2}$	12,000	[12,460, 15,753]

*The units of ${\hat{\gamma }}_{1}$ and ${\hat{\gamma }}_{2}$ are US dollars*.

The threshold regression model was used to study the non-linear relationship between public health investment and industrial structure upgrading. The results shown in Table 6 model (2) indicate that as per capital GDP increases, the impact of public health investment on industrial structure gradually weakens. The impact coefficient of public health investment on industrial structure was −0.126 when per capital GDP was below the first threshold (${\hat{\gamma }}_{1}\leq 4412$). When per capital GDP was in the range [4,412, 12,000], the impact coefficient of public health investment on industrial structure decreased to−0.103. As per capital GDP crossed the second threshold (${\hat{\gamma }}_{1}\geq 12,000$), the impact coefficient was even lower at −0.088, indicating that the effect of public health investment on industrial structure was non-linear. That is, as per capital GDP increased, the crowding out effect of public health investment on industrial structure gradually weakened. The main reasons are as follows. When the economy of an emerging market country is in the initial stage of development, the country is likely to invest heavily in industrial production. Large investments in public health investment have a crowding out effect on the social economy (41, 42). However, when the national economy develops at a higher level, the government has more abundant funds for public health investment, so increases in public health investment will have a smaller crowding out effect on industrial structure upgrading.

**Table 6:** Estimated results of public and private health investment on industrial structure upgrading.

**Variable**	**_Indu_−_Senit_**	**Variable**	**_Indu_−_Senit_**
	**(1)**		**(2)**
*GC*_*it*_	−0.494^***^	*GC*_*it*_	−0.179^***^
	(−7.01)		(−2.63)
*FI*_*it*_	−0.266	*FI*_*it*_	5.52e-12
	(−1.05)		(0.51)
*PR*_*it*_	−0.226^***^	*PR*_*it*_	−0.151^***^
	(−3.44)		(−2.67)
*Hea*_*l*_−_ *Natit*_	−0.126^***^	*Hea*_*l*_−_ *Priit*_	0.061^**^
(*PG*_*it*_ ≤ 4412)	(−3.70)	(*PG*_*it*_ ≤ 4,033)	(2.29)
*Hea*_*l*_−_ *Natit*_	−0.103^**^	*Hea*_*l*_−_ *Priit*_	0.155^***^
(4,412 < *PG*_*it*_ <12,000)	(−0.92)	(4,033 < *PG*_*it*_ <16,000)	(4.91)
*Hea*_*l*_−_ *Natit*_	−0.088^***^	*Hea*_*l*_−_ *Priit*_	1.065^***^
(*PG*_*it*_ ≥ 12,000)	(−2.90)	(*PG*_*it*_ ≥ 16,000)	(13.16)
C	36.19^***^	C	−27.95^***^
	(7.44)		(−4.16)
*N*	361	*N*	361
*R* ^2^	0.619	*R* ^2^	0.748

*** and ** *represent significance levels of 1 and 5% respectively*.

### The Impact of Private Health Investment on the Upgrading of Industrial Structure

A test of the per capital GDP thresholds relevant to private health investment showed that a single threshold effect was significant at the 10% significance level, a double threshold was significant at the 1% significance level, and a triple threshold failed the 10% significance test (Table 7).

**Table 7:** Effect test of private health investment thresholds.

**Model**	**Critical value**					
	***F* value**	***P* value**	**BS times**	**1%**	**5%**	**10%**
Single threshold test	95.162^*^	0.060	300	127.964	97.817	81.731
Double threshold test	337.340^***^	0.000	300	118.140	56.748	30.892
Triple threshold test	0.000	0.285	200	0.000	0.000	0.000

* and *** *are significant at 10 and 1% levels respectively*.

**Figure 3:**
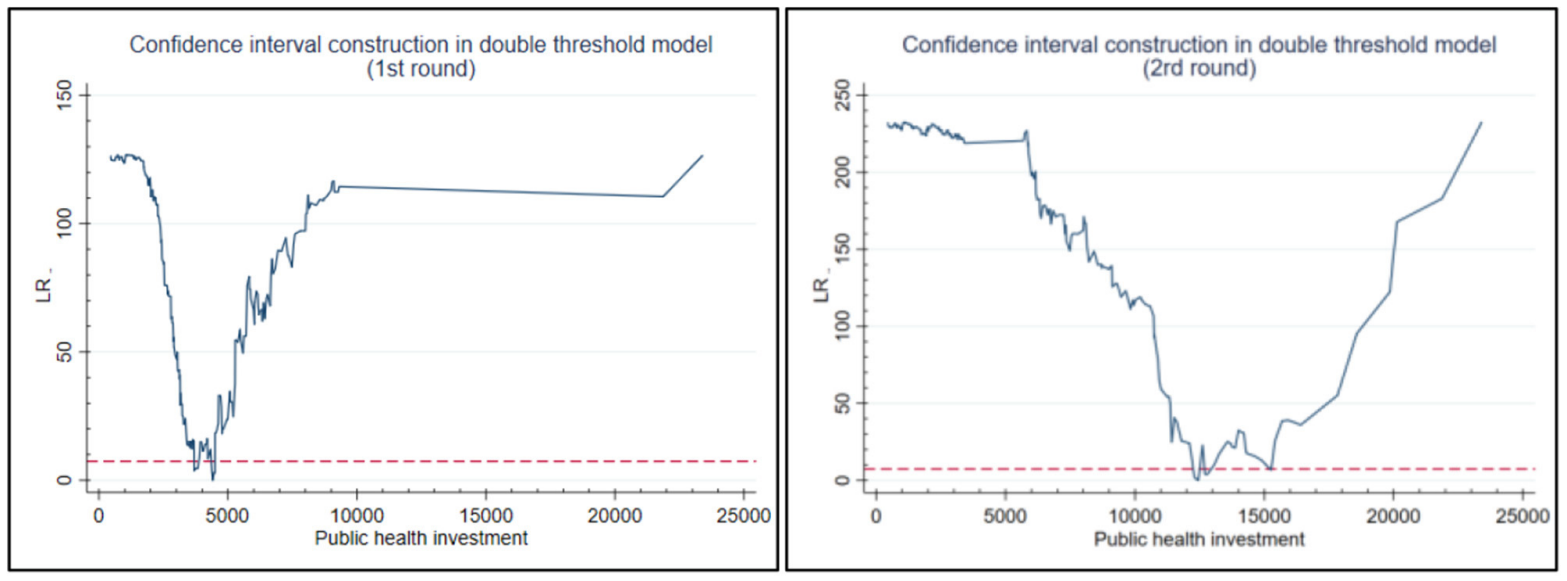
Estimation of the first and second threshold of per capital GDP based on a likelihood ratio function.

The two threshold values of the double threshold model were identified using a likelihood ratio function, as shown in Figure 3. The threshold estimate of per capital GDP (*CS*_*it*_) refers to the value of γ when the likelihood ratio test statistic *LR* is 0. As can be seen from Table 8, the two per capital GDP thresholds were estimated as 4,033 and 16,000 respectively.

**Table 8:** Results of threshold estimation for private health investment.

	**Threshold estimate**	**95% confidence interval**
Threshold value ${\hat{\gamma }}_{1}$	4,033	[3,741, 4,772]
Threshold value ${\hat{\gamma }}_{2}$	16,000	[16,000, 16,500]

*The units of ${\hat{\gamma }}_{1}$ and ${\hat{\gamma }}_{2}$ are US dollars*.

Table 6 model (2) shows that as per capital GDP increased, the impact of private health investment on industrial structure gradually increased. The likelihood ratio test showed that the influence coefficient of private health investment on industrial structure upgrading was positive and significant at the 1% level. That is, the impact of private health investment on industrial upgrading presents a non-linear relationship. Private health investment had a significantly positive impact on industrial structure upgrading when per capital GDP was below the first threshold (${\hat{\gamma }}_{1}$ ≤ 4,033), with an impact coefficient of 0.061. When per capital GDP increased within the range [4033–16000], the effect of private investment on industrial structure upgrading increased significantly, illustrated by a correlation coefficient of 0.154. As per capital GDP crossed the second threshold (${\hat{\gamma }}_{1}$ ≥ 16,000), the impact of private health investment on industrial structure upgrading increased again, albeit by a smaller margin, as illustrated by an impact coefficient of 1.065. These results confirm that private investment has a double threshold effect on industrial upgrading based on per capital GDP level. As the economy develops, the promotion effect of private health investment on industrial structure upgrading gradually increased (43). The possible reasons for this are that private medical services tend to be diverse, flexible, and more effective (44). Therefore, the threshold requirement for per capital GDP level is relatively low. By contrast, public health investment is a large scale investment which requires a higher threshold for per capital GDP before such investments can be made.

**Figure 4:**
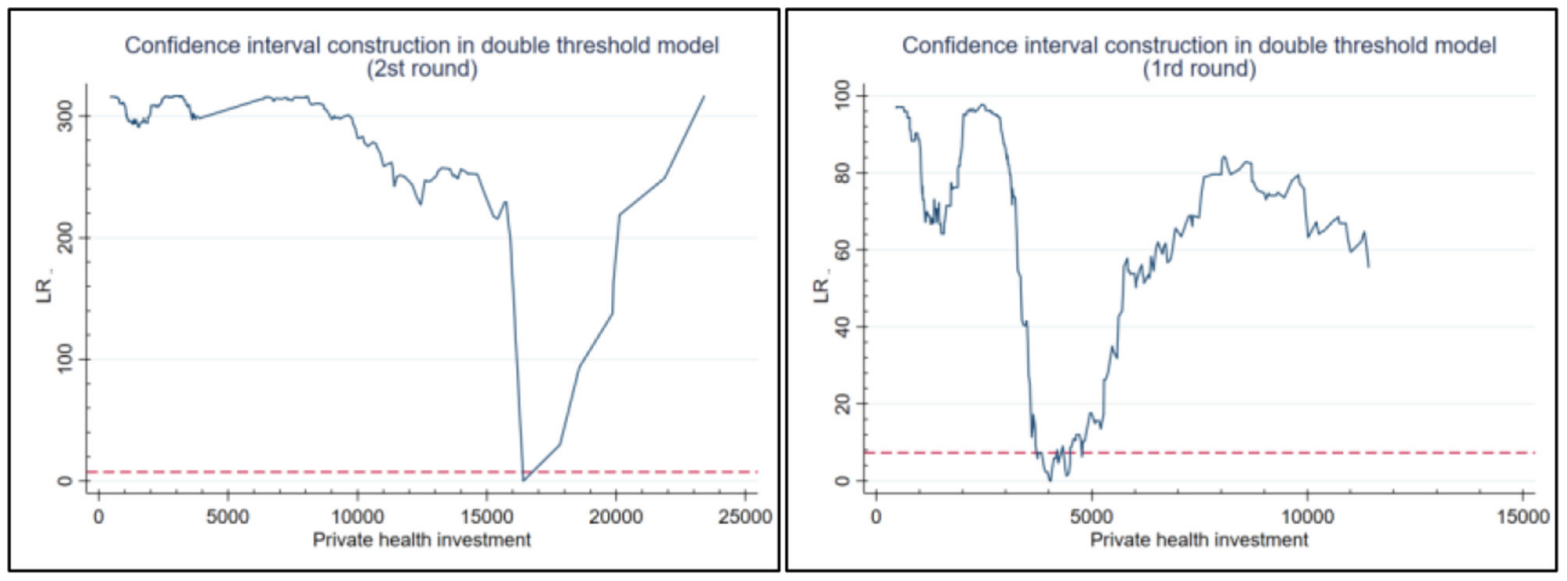
Estimation of the first and second threshold of per capital GDP based on a likelihood ratio function.

Trend lines were then fitted to the relationship between public and private health investment and the level of industrial structure upgrading (Figure 4). (1) These show the change in industrial structure upgrading at the two per capital GDP thresholds (4,412 and 12,000) for public health investment. The per capital GDP thresholds for private health investment were 4,033 and 16,000. The fact that the first per capital GDP threshold for public health investment is higher than it is for private health investment indicates that its promotion effect on industrial structure upgrading should create leapfrog growth and require higher per capital GDP level. However, private health investment has a relatively low requirement on GDP per capita in order to realize leapfrog growth in industrial structure upgrading. However, the promotion effect of private health investment on industrial structure upgrading requires less per capita GDP in order to achieve leapfrog growth. As a government investment, public health investment is large scale, wide ranging and has a clear target (45). Higher per capital GDP levels are necessary to generate enough GDP through taxation to fund such projects.

(2) In terms of impact coefficient, due to the relatively backward economic development in emerging market countries, public health investment has a crowding out effect on the upgrading of industrial structure. However, its crowding out effect on the upgrading of industrial structure is less than the spillover effect of private health investment. Therefore, when making health investment decisions, governments should take account of local conditions and adopt measures that aim to maximize the spillover effect of health investment on industrial upgrading and reduce the crowding out effect.

## Summary and Conclusions

In this paper, health investment was divided into public health investment and private health investment. Using health investment and industrial structure data from emerging markets between 1999 and 2020, fixed effect and threshold regression models were developed to explore the impact of health investment on industrial structure.

The results showed health investment had a significant crowding out effect on primary and secondary industries and a spillover effect on the tertiary industry. Increasing health investment, whether public or private, crowds out other material capital, thus inhibiting economic development. Although public health investment had a crowding out effect on the added value of primary and secondary industries, it nevertheless plays a significant role in the economic growth of emerging market countries by increasing the accumulation of healthy human capital, which is especially important in countries with low fertility rates and aging populations (46). Investment in public health plays an important role in adjusting demographic structure and maintaining human capital (47). In this study, the ratio of employment in the primary, secondary and tertiary industries was used as a proxy variable to assess the robustness of the results. The results showed that the model was robust and reliable.

The crowding out effect of public health investment on primary and secondary industries was shown to be greater than that of private health investment. Moreover, the spillover effect on all three industries was also greater than that of private health investment. This suggests public health investment plays an important role in national health systems in emerging countries (48). When public health investment, governments is increased modestly, governments should encourage private investment and promote the growth of tertiary industry, to reduce the crowding out effect of public health investment on primary and secondary industries. Given the limited nature of governmental annual investment, increases in health investment will inevitably reduce investment in the primary and secondary industries, thus inhibiting the output of the primary and secondary industries.

**Figure 5:**
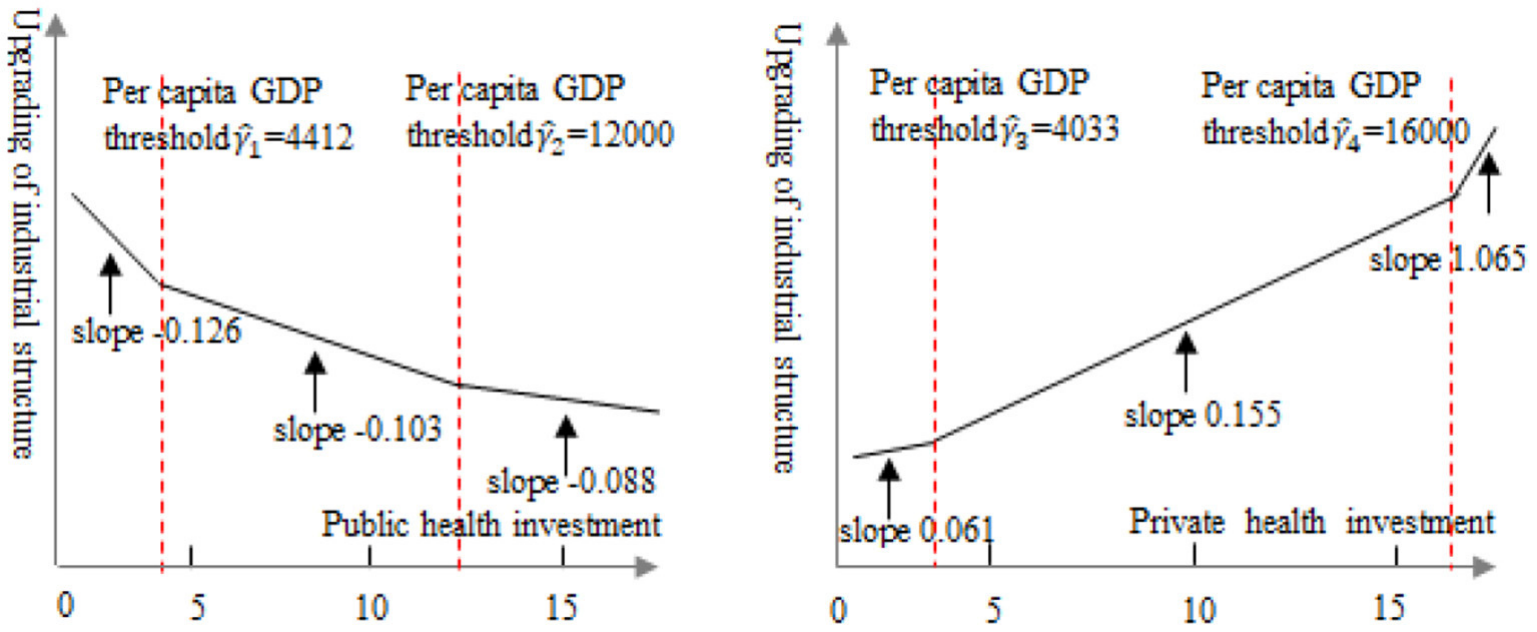
Trend line illustrating the relationship between public (left) and private (right) health investment and industrial structure upgrading.

The study also demonstrated the non-linear relationship between public and health investment and industrial structure upgrading. As per capital GDP of emerging market countries increases year by year, its inhibiting effect on industrial structure upgrading gradually weakens. The promotion effect of private health investment on industrial structure upgrading gradually increases, and the two together form a U-shaped curve (Figure 5). To achieve leapfrog growth of industrial structure upgrading, private health investment have a low requirement on the first threshold value of industrial per capital GDP. For emerging market countries, increasing health investment can promote industrial structure upgrading by improving human capital. However, in emerging market countries with low levels of economic development, high levels of health investment will crowd out investment in other fields, which may hinder industrial structure upgrading (49, 50). The net effect of health investment on economic growth depends on the size of these two mechanisms.

Based on the results of this study the following conclusions were drawn. Firstly, health investment has a “double-edged sword” effect on the upgrading of industrial structure. For developed countries with well-developed medical systems, increasing health investment improves the overall health of the population and contributes to increases in national wealth and living standards. However, in less economically developed countries, public and private health investment is an economic burden to government and families and may hinder national economic growth and improved living standards. Secondly, the role of public health investment in industrial structure upgrading should not be ignored. As health investment crowds out physical capital investment, economic growth will not occur in the short term. However, in the long term, health investment contributes to the development of a healthy labor force, thus promoting economic growth. Therefore, while health investment can hinder the upgrading of industrial structure in the short term, it can promote it over the long term. Thirdly, governments of emerging market countries should seek to identify the “critical point” at which health investment maximizes economic growth. Under different per capital GDP levels, public health investment and private health investment have different effects on industrial structure upgrading. Emerging market countries typically have a low level of economic development, so governments should provide incentives and support in tax and industrial policies to attract private health investment, so as to relieve the financial pressure on the government. Government spending can be invested elsewhere and in so doing may avoid a negative impact on the economy.

Based on the above findings, the following policy implications were derived. (1) The government should introduce relevant policies to encourage private health investment and improve the level of private health investment. Both the existing research and the empirical conclusion of this paper prove that private health investment contributes to the upgrading of industrial structure (51). Therefore, the government should strengthen the publicity and education of health awareness and popularize the awareness of health investment. At the same time, the government has given policy support to private health and medical investment, actively advocated the development of health industry, and made people realize the significance of private health investment. (2) The government has improved the basic medical and health care system to ensure human resources for economic development. While speeding up economic development, emerging market countries should give top priority to people's health, establish a private medical and health care system at night and a major epidemic emergency management system to ensure people's health, provide human resources for economic development, and promote economic development and industrial structure upgrading. (3) The government should accelerate the transformation of economic growth pattern, that is, from traditional economic growth to healthy economic growth. Although public health investment has a “short-term effect” on healthy economic growth, in the long run, health investment has a promoting effect on economic growth and industrial structure upgrading. At present, emerging market countries are still in the transition from traditional economic growth mode to healthy economic mode. Increasing health investment is the most basic and important way to realize healthy economic growth. Therefore, the government should accelerate investment in health and promote the transformation of economic growth pattern.

This study had some limitations, (1) Lack of discussion of the spatial dimension. The impact of health investment on industrial structure upgrading involves time and space dimensions. However, this study only discussed from time dimension, and lacked research on spatial autocorrelation and adjacency. Health investment may have spatial siphon effect and spatial spillover effect on industrial structure upgrading. (2) The calculation of industrial structure upgrading needs further study. Due to inconsistent statistics and data deficiency from emerging market countries, the ratio of the added value of the tertiary industry to the added value of the secondary industry is used as the index of industrial structure upgrading. However, we should use a variety of measurement methods to calculate the upgrading of industrial structure to test the robustness of the results.

## Data Availability Statement

Publicly available datasets were analyzed in this study. This data can be found here: https://www.worldbank.org/en/home.

## Author Contributions

QC: conceptualization, methodology, and software. QC and H-MW: data curation and writing—original draft preparation. H-MW and Y-PZ: visualization and investigation. QC and Y-PZ: writing—reviewing and editing. All authors contributed to the article and approved the submitted version.

## Funding

This study was supported by the National Natural Science Foundation of China (41871137 and 41867071), the Scientific Research Cultivation Project 2020 of the Hubei University of Economics (PYYB202009), the Wuhan Social Science Federation (WHSKL2021149), General Project of Philosophy and Social Science Planning of Anhui Province (AHSKY2018D17), and the Scientific Research Project of Hubei Education Department (B2021171).

## Conflict of Interest

The authors declare that the research was conducted in the absence of any commercial or financial relationships that could be construed as a potential conflict of interest.

## Publisher's Note

All claims expressed in this article are solely those of the authors and do not necessarily represent those of their affiliated organizations, or those of the publisher, the editors and the reviewers. Any product that may be evaluated in this article, or claim that may be made by its manufacturer, is not guaranteed or endorsed by the publisher.
